# Genome-Wide Methylation Changes Associated with Replicative Senescence and Differentiation in Endothelial and Bone Marrow Mesenchymal Stromal Cells

**DOI:** 10.3390/cells12020285

**Published:** 2023-01-11

**Authors:** Angelica Giuliani, Maria Giulia Bacalini, Deborah Ramini, Emanuela Mensà, Chiara Giordani, Luciano Xumerle, Paolo Garagnani, Fabiola Olivieri, Antonio Domenico Procopio, Maria Rita Rippo, Jacopo Sabbatinelli

**Affiliations:** 1 Department of Clinical and Molecular Sciences, Università Politecnica Delle Marche, 60126 Ancona, Italy; 2 IRCCS Istituto delle Scienze Neurologiche di Bologna, 40139 Bologna, Italy; 3 Clinic of Laboratory and Precision Medicine, IRCCS INRCA, 60121 Ancona, Italy; 4 Personal Genomic S.R.L, 37134 Verona, Italy; 5 Department of Experimental, Diagnostic and Specialty Medicine, University of Bologna, 40126 Bologna, Italy; 6 Applied Biomedical Research Center (CRBA), S. Orsola-Malpighi Polyclinic, 40126 Bologna, Italy; 7 CNR Institute of Molecular Genetics “Luigi Luca Cavalli-Sforza”-Unit of Bologna, 40126 Bologna, Italy; 8 Department of Laboratory Medicine, Clinical Chemistry, Karolinska Institutet, Karolinska University Hospital, 141 86 Huddinge, Sweden; 9 Laboratory Medicine Unit, Azienda Ospedaliero Universitaria delle Marche, 60126 Ancona, Italy

**Keywords:** DNA methylation, bone-marrow mesenchymal stromal cells, endothelial cells, adipocytes, osteoblasts, cellular senescence

## Abstract

Bone marrow mesenchymal stromal cells (BMSCs) are multipotent cells able to self-renew and differentiate, depending on the microenvironment, into adipocytes and osteoblasts. These cells have a limited number of replications and enter replicative senescence during in vitro expansion. The role of DNA methylation (DNAm) assumes importance in cell function and commitment; however, its exact contribution to BMSC differentiation and replicative senescence is still unclear. We performed a genome-wide DNAm analysis on BMSCs cultured in vitro at early passages and induced to differentiate into adipocytes and osteoblasts, and on replicative senescent BMSCs and HUVECs, to identify DNAm patterns of senescence and differentiation. We also compared BMSCs and HUVECs in replicative senescence and found that, in both cellular systems, genome-wide hypomethylation was accompanied by a higher-than-expected overlap of differentially methylated positions (DMPs) and concordance in terms of direction of the change. A Kyoto Encyclopedia of Genes and Genomes (KEGG) pathway enrichment analysis on lineage-independent senescence-associated DMPs revealed 16 common pathways, including Insulin resistance, Molecule adhesion, and Wnt/β-catenin signaling. In both adipogenesis and osteogenesis, we observed a general demethylation of CpG sites compared with undifferentiated BMSCs with a higher number of DMPs in osteogenesis. KEGG analysis resulted in 30 pathways enriched in osteoblasts and only 2 in adipocytes when compared to undifferentiated cells. When comparing differentiated BMSCs with senescent ones, osteogenesis exhibited a greater overlap with senescence in terms of number of DMPs and direction of methylation change compared to adipogenesis. In conclusion, this study may be useful for future research on general mechanisms that occur in replicative senescence and furthermore to identify trajectories of BMSC differentiation and common aspects of differentiated and senescent cells.

## 1. Introduction

Bone marrow (BM)-derived mesenchymal stromal cells (BMSCs) are self-renewing cells with the potential to differentiate into multilineage precursors. The fate destination of BMSCs, i.e., the self-renewal division and the choice of differentiation program into adipocytes or osteoblasts, is tightly regulated by numerous stimuli to which the cells are exposed in the bone-marrow milieu [1]. Numerous evidence showed how osteogenic and adipogenic differentiations exhibit reciprocal and competing features: in fact, pro-adipogenic induction factors inhibit osteogenesis and vice versa [2,3]. Thus, the loss of balance in these finely regulated programs can lead to an abnormal differentiation ultimately causing changes in the BM fat volume and bone mineral density, which are associated with several pathological conditions, including among others osteoporosis, anorexia nervosa, multiple myeloma and aging in general [4]. Furthermore, some evidence suggests that BMSCs undergo senescence during organismal aging, with a reduced proliferation capacity [5,6] exacerbating bone loss and reducing the regenerative capacity of tissues.

Due to their emerging roles not only in metabolic diseases, but also in age-related diseases (ARDs), the commitment of MSCs in bone marrow has attracted significant attention. The lineage commitment of MSCs can be induced by different cues, including chemical (such as component used for in vitro commitment, i.e., indomethacin, dexamethasone, insulin, ascorbic acid) physical (cell shape, external mechanical forces, extracellular matrix), and biological factors (aging, metabolism) [1]. In vitro MSCs without any differentiating stimuli undergo a series of replications up to a state of replicative senescence, characterized by a stable arrest of growth and a compromised state of regenerative and stemness capacity [7]. Indeed, MSCs obtained from older donors show an impaired proliferation and differentiation capacity compared to those of younger donors [8].

Among the biological mechanisms involved in determining cell fate, epigenetic alterations to DNA and chromatin have been shown to impact both differentiation and senescence [9,10,11]. In mammals, the addition of a methyl group at the 5-position of the cytosine (C) residue occurs mostly within cytosine-guanine dinucleotides (CpG sites) and it is catalyzed by DNA methyltransferases (DNMTs). Age-associated alterations in DNA methylation (DNAm) are characterized by a gradual and extensive genome-wide hypomethylation alongside with hypermethylation of specific genomic regions, including promoter-associated CpG islands [12]. This senescence-associated differential methylation pattern has been observed in cultured fibroblasts, endothelial cells, and MSCs [13,14].

DNAm also plays a pivotal role in MSC commitment towards osteogenic lineage [15], whereas its role in the adipogenic one is still not clear [16]. A number of candidate genes affecting the fate and function of MSCs have been shown to be tightly regulated at the epigenetic level and proposed as therapeutic targets for diseases associated with alterations in MSC differentiation [15,17]. While reports have been published on the epigenetic signatures driving MSC commitment, the comparison of the methylation profile between BMSC-derived adipocytes and osteoblasts and, furthermore, between these terminally differentiated cells and senescent ones has not been explored to date.

Therefore, the aim of our study was to analyze the changes in DNAm of human-derived (h-)BMSCs committed to differentiate into adipocytes and osteoblasts and in replicative senescence. We also wanted to evaluate a possible common or cell-type specific DNAm signature in the establishment of replicative senescence by comparing hBMSCs with the well-characterized model of human umbilical vein endothelial cells (HUVECs) [18]. This analysis would allow us to unravel divergent and/or common epigenetic signatures still unknown and to deepen the knowledge of those epigenetic mechanisms that cause alteration or degeneration of the bone, especially during human aging.

## 2. Materials and Methods

### 2.1. Cell Lines and Cell Culture

Human bone marrow-derived mesenchymal stem cells (BMSCs) were purchased from Lonza (Catalog PT-2501, donor #110877, 22-year-old male) and maintained in α-MEM medium supplemented with 10% of fetal bovine serum (FBS), 1% of L-glutamine and 1% of Penicillin/Streptomycin. Cryopreserved primary human umbilical vein endothelial cells (HUVECs) were purchased from Clonetis (Lonza, Basel, Switzerland) and cultured in Endothelial Basal Medium (EBM-2, CC-3156, Lonza) supplemented with SingleQuot Bullet Kit (CC-4176, Lonza). Cells were maintained at 37 °C a humidified atmosphere containing 5% CO_2_, seeded at a density of 5000/cm^2^ in T75 flasks (Corning Costar, Sigma Aldrich, St. Louis, MO, USA), with a change of medium every 48 h. Upon reaching 80% of confluence, cells were washed with PBS, detached with 0.25% trypsin -EDTA (ECB3052; EuroClone, Milano, Italy), and passaged.

For the establishment of replicative senescence, BMSCs and HUVECs were cultured until they arrested their replication (approximatively around the 17th passage), and replicative senescence was assessed using Senescence-Associated (SA)-β-galactosidase and measuring the Population Doublings (PDs). PDs were calculated by the formula: (log10(F) − log10(I))/log10(2), where F is the number of cells at the end of the passage, and I is the number of seeded cells. Cumulative population doublings (cPD) were calculated as the sum of PD changes. HUVECs were considered at early passages for passages ≤6 and senescent at passage ≥16; BMSCs were considered young at passage ≤5 and senescent at passage ≥14.

For adipogenic and osteogenic differentiation, BMSCs were seeded at 8 × 10^3^ cells/cm^2^ on six-well plates in adipogenic medium containing complete α-MEM, 2 mM L-glutamine, 100 U/mL penicillin, 100 mg/mL streptomycin, and 10% FBS supplemented with 0.5 μM dexamethasone, 5 μg/mL insulin, 0.2 mM indomethacin, and 0.45 mM isobutylmethylxantine (Sigma-Aldrich) [19]. For osteogenic differentiation, BMSCs were seeded at 6 × 10^3^ cells/cm^2^ on six-well plates in osteogenic medium containing complete α-MEM, 2 mM L-glutamine, 100 U/mL penicillin, and 100 mg/mL streptomycin, and 10% FBS supplemented with 0.2 mM L-ascorbic acid, 10 mM β-glycerophosphate, and 0.1 μM dexamethasone (Sigma-Aldrich). The adipogenic/osteogenic medium was changed twice a week until the end of the experiment (15 days).

### 2.2. Senescence-Associated β-Galactosidase Staining

Senescence-associated expression of Beta-Galactosidase (β-Gal) activity was detected using Senescence Detection Kit (BioVision Inc., Milpitas, CA, USA) according to the manufacturer’s protocol. Briefly, non-confluent HUVECs and BMSCs cultured in 24-well plates were fixed for 15 min at room temperature. After two washes with PBS, cells were incubated overnight at 37 °C with a solution containing X-Gal, i.e., the substrate of β-gal. When X-gal was hydrolyzed by the enzyme, it was transformed into an insoluble blue pigment which was visible in the cell cytoplasm by light microscopy. The percentage of positive cells was determined by counting at least 500 cells/well.

Cells were considered “early passage” when SA-β-Gal < 10% and “senescent” (Sen) when SA-β-Gal > 80%.

### 2.3. Alizarin Red Staining

The Alizarin Red stain (ARS) for calcium deposits is commonly used to detect mineral deposits in osteoblast differentiating cells. Briefly, BMSCs were fixed with paraformaldehyde 4% for 15 min and, after three washes with deionized water, were incubated with ARS for 30 min with gentle shaking. The cells were washed with deionized water and. ARS-stained cells can be visualized through light microscopy.

### 2.4. Oil Red O Staining

Adipocyte differentiation was assessed by Oil Red O (ORO) staining. Briefly, cells were washed with PBS and fixed with 4% paraformaldehyde for 5 min. BMSCs were washed twice with PBS and then incubated with freshly filtered ORO staining solution (six parts Oil Red O stock solution and four parts H_2_O; Oil Red O stock solution is 0.5% Oil Red O in isopropanol) for 30 min. ORO staining can be visualized through light microscopy.

### 2.5. Analysis of mRNA Expression

Total RNA was extracted from HUVECs and hBMSCs using Norgen total RNA Purification Kit (cat. no. 37500, Norgen Biotek Corporation, Thorold, Canada) according to the manufacturer’s protocol. Purified RNA was stored at −80 °C until analysis. Next, 1 μg of purified RNA was retrotranscribed using PrimeScript™ RT reagent Kit with gDNA Eraser (Perfect Real Time) (Takara, Kusatsu, Japan). Then, Real Time PCR reactions were conducted in a Rotor Gene Q 5plex HRM apparatus (Qiagen, Hilden, Germany) in a 10 μL total reaction volume using TB Green Premix Ex Taq II (Takara), according to the manufacturer’s instructions. The mRNA expression of the genes of interest was calculated with the reference genes β-actin. mRNA expression levels were analyzed by the 2^−ΔCt^ method. The value of the relative expression of the genes of interest is given as the mean ± standard deviation (SD) of three independent experiments. Primers used are (5′-3′): p16 (*INK4A*), Fw: CATAGATGCCGCGGAAGGT, Rv: CTAAGTTTCCCGAGGTTTCTCAGA; *PPARG*, Fw: AGCCTCATGAAGAGCCTTCCA, Rv: ACCCTTGCATCCTTCACAAGC; *FABP4*, Fw: TCACCTGGAAGACAGCTCCT, Rv, AAGCCCACTCCCACTTCTTT; *ADIPOQ*, Fw: CCTAAGGGAGACATCGGTGA, Rv: GTAAAGCGAATGGGCATGTT; *RUNX2*, Fw: AGATGGGACTGTGGTTACTG, Rv: TAGCTACTTGGGGAGGATT; *BMP2*, Fw: GGAATGACTGGATTGTGGCT, Rv: TGAGTTCTGTCGGGGACACAG; *OCN*, Fw: ACCTGTATCAATGGCTGGGAG, Rv: TCAGCCAACTCGTCACAGTC and β-actin, Fw: AAACTGGAACGGTGAAGGTG, Rv: CAAGGGACTTCCTGTAACAATGC.

### 2.6. Genome-Wide DNA Methylation Analysis

Genomic DNA was extracted in triplicate from early and Sen BMSCs and HUVECs and from BMSC-derived adipocytes and osteoblasts using Qiagen’s QiAmp mini kit following the manufacturer’s recommendations. Next, 1 μg DNA was bisulfite-converted using EZ DNA Methylation (Zymo Research, Irvine, CA, USA) and analyzed by the Infinium Human MethylationEPIC BeadChip (Illumina Inc., San Diego, CA, USA) according to the manufacturer’s instructions. Raw data were extracted using minfi R package [20] and normalized using the preprocessFunnorm function. Not-reliable probes according to Zhou and collaborators [21] and probes on X and Y chromosomes were removed. The remaining 748,241 probes were annotated according to IlluminaHumanMethylationEPICanno.ilm10b4.hg19 annotation data.

### 2.7. Pathway Enrichment Analysis

Kyoto Encyclopedia of Genes and Genomes (KEGG) enrichment analyses was performed using the methylgometh function in the methylGSA R package [22] by taking into account that the number of CpGs assigned to each gene differs by accounting for the probability of a gene being selected using Wallenius’ noncentral hypergeometric distribution. All tested probes were used as background, and the *p*-values were adjusted using the Benjamin–Hochberg method.

### 2.8. Statistical Analysis of Data

The Student’s t test was applied to determine statistically significant between samples. *p*-Values less than 0.05 were considered significant. Differences in methylation between non senescent and senescent cells were analyzed applying Wilcoxon sign ranks, after Bonferroni’s correction. The R package limma [23] was used to find differentially methylated positions (DMPs); the *p*-values resulting from each comparison were adjusted for multiple tests using the Benjamini–Hochberg (BH) procedure. Enrichment of DMPs across genomic regions (islands, N- and S-shores and shelves, open sea regions) was calculated using the fisher.test function from the stats R package (*p*-value < 0.05). The same function was used to evaluate whether the overlap between DMPs resulting from different comparisons was higher than expected. All the analyses were performed using R version 3.6.3.

## 3. Results

### 3.1. Experimental Design and Characterization of the Cellular Model

**Figure 1:**
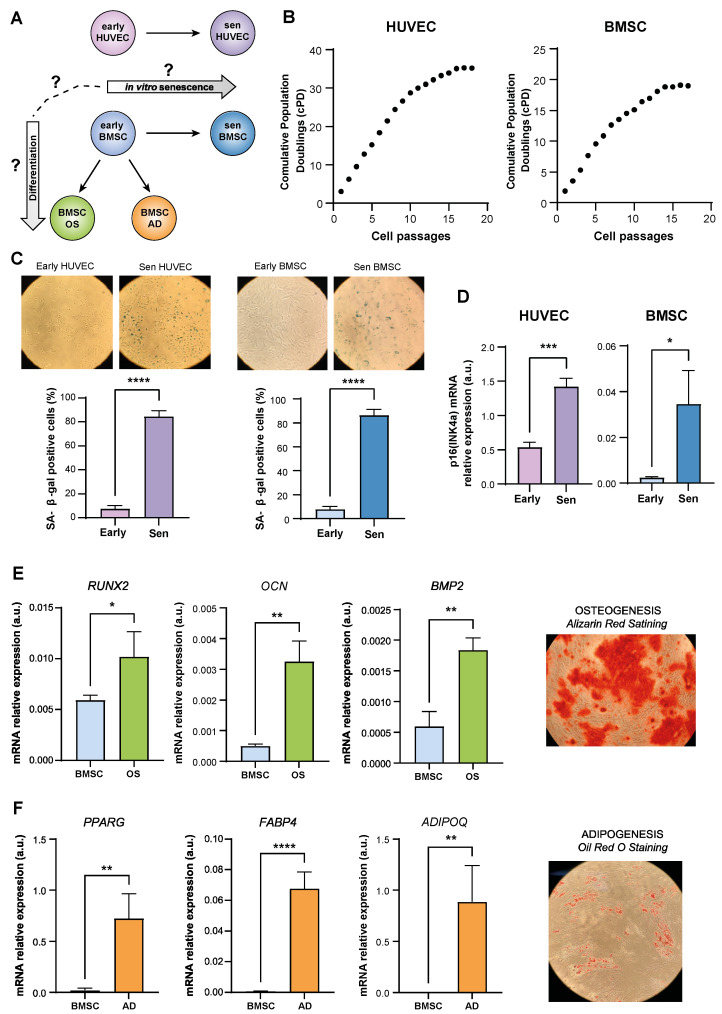
Experimental design and characterization of cellular models used for genome-wide DNAm analysis. (**A**) Experimental design: early-passage HUVECs and BMSCs were cultured until they reached replicative senescence (Sen); BMSCs were induced to differentiate into adipocytes (AD) and osteoblasts (OS) for 15 days. (**B**) Growth curve showing Cumulative Population Doublings (CPDs) of BMSCs and HUVECs. (**C**) Representative images and quantification of the SA β-Gal staining positivity in early (SA β-Gal < 10%) and senescent (SA β-Gal > 80%) HUVECs and BMSCs. (**D**) p16(INK4a) relative expression in arbitrary units (a.u.) in early and senescent cells obtained through Real Time PCR. Data were normalized using β-actin as internal control. (**E**) Runx2, Osteocalcin (OCN), and Bone morphogenetic protein 2 (BMP-2) relative expression (a.u.) and Alizarin red staining were used to verify the osteogenic differentiation (OS). (**F**) PPARγ, Adiponectin (ADIPOQ) and Fatty Acid Binding Protein 4 (FABP4) mRNA relative expression and Oil Red O staining were used to verify the adipogenic differentiation (AD). Data were normalized using β-actin as internal control. Data are the mean ± S.D. of three independent experiments. *, *p* < 0.05; **, *p* < 0.01; ***, *p* < 0.001, ****, *p* < 0.0001 for paired *t*-test.

To uncover DNAm patterns associated with replicative senescence and BMSC differentiation, genome-wide DNAm was analyzed in BMSCs and HUVECs at early passages and late passages and in BMSCs induced to differentiate into osteoblasts (OS) and into adipocytes (AD) (Figure 1A). The achievement of senescence of HUVECs and BMSCs was characterized by analyzing growth arrest, documented by reduced cumulative population doublings (cPDs) (Figure 1B), and assayed by Senescence Associated-β-galactosidase (SA-β-gal) activity (Figure 1C) and upregulation of the cell cycle regulators p16(INK4a) mRNAs (Figure 1D). BMSC osteogenic differentiation was evaluated by expression analysis of the key transcription factor associated with osteoblast differentiation Runt-related transcription factor 2 (*RUNX2*), the osteogenesis-related proteins Bone morphogenetic protein 2 (*BMP2*) and osteocalcin (*OCN*); alizarin red staining was performed as further confirmation of a successful osteogenesis (Figure 1E). Similarly, adipogenic differentiation was evaluated by expression analysis of the key transcription factor associated with adipocyte differentiation PPARγ, Fatty Acid Binding Protein 4 (*FABP4*) and Adiponectin (*ADIPOQ*), and Oil Red O for fat-soluble staining (Figure 1F).

### 3.2. Genome-Wide DNAm across Cell Type, Differentiation, and Senescence

**Figure 2:**
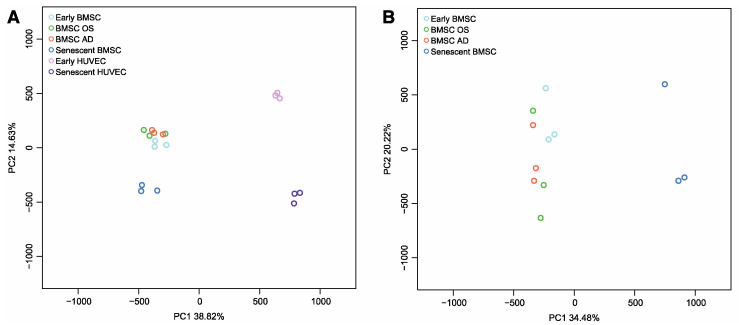
Principal Component Analysis (PCA) of DNAm values obtained by genome-wide analysis (**A**) in early and senescent HUVEC and in early, senescent, and differentiated BMSC samples, and (**B**) in early, senescent, and differentiated BMSC samples (*n* = three replicates per condition). The percentage of variance explained by the first and the second principal components is reported in X and Y axes, respectively.

After data pre-processing (see Materials and Methods), we used Principal Component Analysis (PCA) to explore the main sources of variability in genome-wide DNAm across cell type, differentiation, and senescence status (Figure 2). As expected, PC1, which explained 38.82% of total variance, separated samples according to cell type, with BMSCs and HUVECs clearly distinguished. In PC2 (14.63% of total variance) both BMSCs and HUVECs separated according to senescence status (Figure 2A). When only BMSCs were considered, the separation between senescent and early passage or differentiated BMSCs was confirmed, whereas a less evident segregation between early passage and differentiated BMSCs was present along PC2, suggesting that BMSC senescence is accompanied by more pronounced DNAm changes compared to differentiation (Figure 2B).

Distribution of DNAm values in the samples showed a clear hypermethylation of HUVECs with respect to BMSCs (Figure S1A). To further explore these widespread DNAm differences, we evaluated the distribution of DNAm dividing the probes according to their localization near/within genic sequences and/or CpG-rich regions (Materials and Methods; Figure S1B). We found that HUVEC-associated hypermethylation was mainly driven by probes mapping in genomic regions that are neither associated to genes nor to CpG-rich sequences. Moreover, the same analysis showed that for both BMSCs and HUVECs, senescent cells tended to be hypomethylated with respect to the early-passage counterpart, especially for probes mapping in CpG-rich regions not associated to genes and in open sea regions (Figure S1B).

### 3.3. DNAm Changes Associated with Senescence in BMSCs and HUVECs

**Figure 3:**
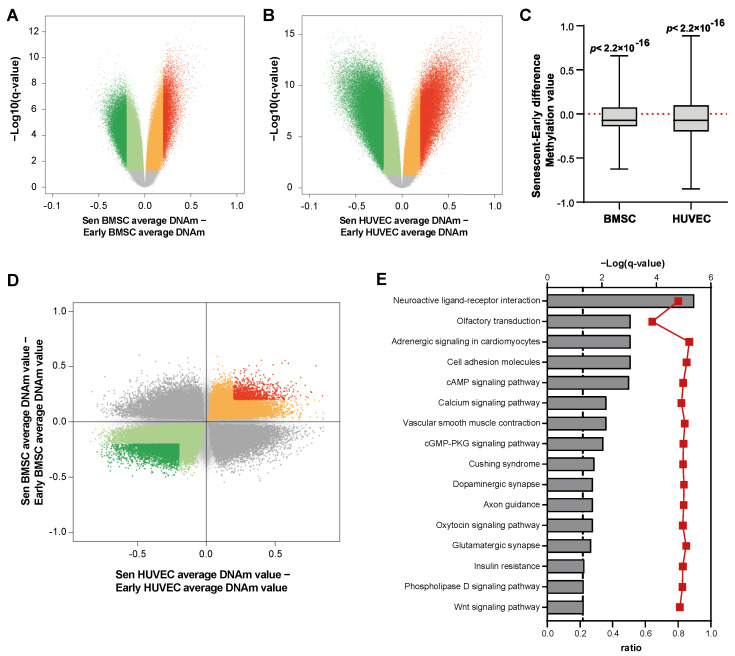
(**A**,**B**) Volcano plot showing differentially methylated CpGs (FDR < 0.05) in (**A**) Senescent (Sen) vs. early BMSCs and (**B**) Sen vs. early HUVECs. Differences in DNAm values are plotted against −log_10_(q-values). DMPs with an absolute DNAm difference >0.20 are highlighted in red (hypermethylated) and dark green (hypomethylated). (**C**) Boxplots showing the distribution of differences in the DNAm values of the CpGs between senescent and early BMSCs and HUVECs. *p* for Wilcoxon signed-rank tests. (**D**) Scatter plot comparing DNAm differences between Sen and early HUVECs and BMSCs. DMPs (FDR < 0.05) with concordant senescence-associated hypo- and hyper-methylation are indicated in green and red, respectively; DMPs with an absolute DNAm difference >0.20 are highlighted with darker green and red colors. (**E**) KEGG pathways significantly enriched in genes containing DMPs that are common to both cellular lineages in senescence. Pathways are ranked according to the significance of enrichment (grey bars, upper *y*-axis). Ratios referring to the proportion of targeted genes related to the total number of genes in each pathway are displayed (red line graph, bottom *y*-axis).

We first searched for differentially methylated positions (DMPs) associated to senescence in BMSCs and HUVECs. Out of 748,241 probes, we identified 240,111 DMPs when comparing late- and early-passage BMSCs and 263,179 DMPs when comparing late- and early-passage HUVECs (BH-corrected *p*-value < 0.05; Figure 3A,B and Figure S2A,B; Supplementary Files S1 and S2). In both cells types the majority of DMPs were hypomethylated in senescent cells (63.4% in BMSCs, 56.5% in HUVECs). This trend towards hypomethylation was even more pronounced when we considered DMPs with an absolute mean DNAm difference between senescent and young cells larger than 0.2: according to these more restrictive criteria we found 35,302 DMPs in MSCs, 72% of which were hypomethylated, and 94,164 DMPs in HUVECs, 71% of which were hypomethylated. This pattern of hypomethylation was further confirmed by multiple one-sample Wilcoxon tests showing that the median of DNAm differences across differentially methylated DMPs was significantly smaller than 0 (null hypothesis) (Figure 3C). Notably, a greater heterogeneity in terms of magnitude of senescence associated DNAm changes was observed for HUVECs compared to BMSCs.

BMSCs and HUVECs showed a similar pattern of enrichment of DMPs across genomic features, characterized by an underrepresentation of CpG islands and a marked enrichment of open sea regions (i.e., genomic regions not rich in CpGs) (Figure S3).

A substantial number of probes (122,303) was found differentially methylated in both the comparisons; the extent of these intersection dropped down to 8108 when we considered the more stringent list of DMPs with an absolute mean DNAm difference between senescent and young cells larger than 0.2. Finally, we considered the concordance of DNAm change (that is, hypermethylation or hypomethylation) of the DMPs in common between MSCs and HUVECs. For both the full and the restricted lists of DMPs, MSCs and HUVECs displayed a high concordance in probes hypomethylated in senescence (Figure 3D).

A KEGG pathway enrichment analysis was conducted on the DMPs in HUVECs and BMSCs, and on the probes that were differentially methylated in the replicative senescence of both cell lineages. The results (Supplementary File S3) show the enrichment of 38 pathways associated with DMPs in senescent HUVECs, 23 pathways in senescent MSCs (Supplementary File S3), and 16 pathways that are common to both lineages (Figure 3E), including the Cell adhesion molecules, Insulin resistance, and Wnt signaling pathways.

### 3.4. DNAm Changes Associated with Osteogenic and Adipogenic Differentiation of BMSCs

We next analyzed our dataset to identify DMPs between undifferentiated BMSCs, BMSCs differentiated into osteoblasts and adipocytes.

**Figure 4:**
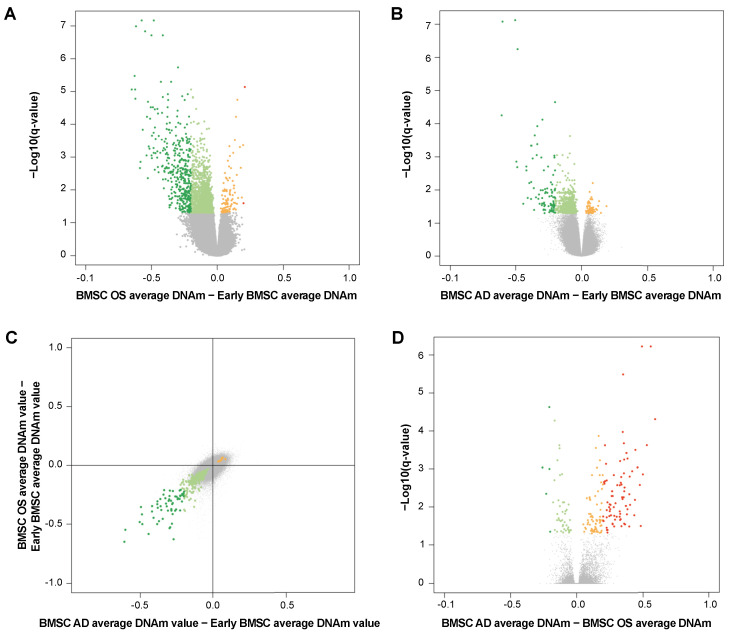
(**A**,**B**,**D**) Volcano plot showing differentially methylated CpGs (FDR < 0.05) in (**A**) osteoblasts (OS) vs. early BMSCs, (**B**) adipocytes (AD) vs. early BMSCs, (**D**) AD vs. OS. Differences in DNAm scores are plotted against −log_10_(q-values). DMPs with an absolute DNAm difference are highlighted in dark red (hypermethylated) and dark green (hypomethylated). (**C**) Scatter plot comparing DNAm differences associated to adipogenic differentiation (*x*-axis) and osteogenic one (*y*-axis). DMPs (FDR < 0.05) with hypo- and hyper-methylation in both the comparisons are indicated in green and red, respectively; DMPs with an absolute DNAm difference >0.20 are highlighted with darker green and red colors.

The comparison between BMSC-derived osteoblasts and undifferentiated BMSCs returned 2319 DMPs (BH-corrected *p*-value < 0.05), 95% of which were hypomethylated. In total, 388 DMPs satisfied the more stringent criteria of an absolute mean methylation difference of 0.2, and all but 2 of them were hypomethylated in differentiated cells with respect to undifferentiated MSCs (Figure 4A and Figure S4A; Supplementary File S4). Pathway analysis on the DMPs revealed a significant enrichment of 30 KEGG terms, including the Wnt signaling pathway, which showed the highest score (q-value = 0.003, Supplementary File S3).

Similar results were obtained for the BMSC-derived adipocytes. In the comparison with undifferentiated MSC we identified 751 and 91 DMPs, using, respectively, the less and more stringent criteria; in both cases, almost the totality of probes was less methylated in differentiated cells compared to undifferentiated BMSCs (Figure 4B and Figure S4B; Supplementary File S5). The pathways showing the highest enrichment in genes with DMPs were the Inositol phosphate metabolism, Phoshatidylinositol signaling system, and axon guidance pathways (Supplementary File S3).

DMPs showed a similar genomic distribution in osteoblasts and adipocytes, with a significant enrichment of hypomethylation in open sea regions (Figure S5).

The intersection between the comparisons was higher than expected (Fisher exact test *p*-value <0.05) and returned 374 probes (59 when considering the more stringent criteria). Notably, all the shared DMPs displayed the same direction of DNAm change in differentiated with respect to undifferentiated MSCs (366 hypomethylated and 8 hypermethylated in both osteogenic and adipogenic differentiation (Figure 4C; Supplementary Files S4 and S5) and were associated with a significant enrichment of the Inositol phosphate metabolism (combined score = 86.85, q-value = 0.002) and Phosphatidylinositol signaling system (combined score = 71.28, q-value = 0.010) pathways.

Then, we compared DNAm of osteoblast and adipocytes. This comparison yielded 197 DMPs, about half of which (86) displayed absolute DNAm changes higher than 0.2 (Figure 4D; Supplementary File S6). Most of these DMPs were more methylated in BMSCs differentiated in adipocytes with respect to osteoblasts and mainly mapped in not CpG-rich regions (Figure S5).

### 3.5. DNAm Relationship between Senescence and Differentiation

**Figure 5:**
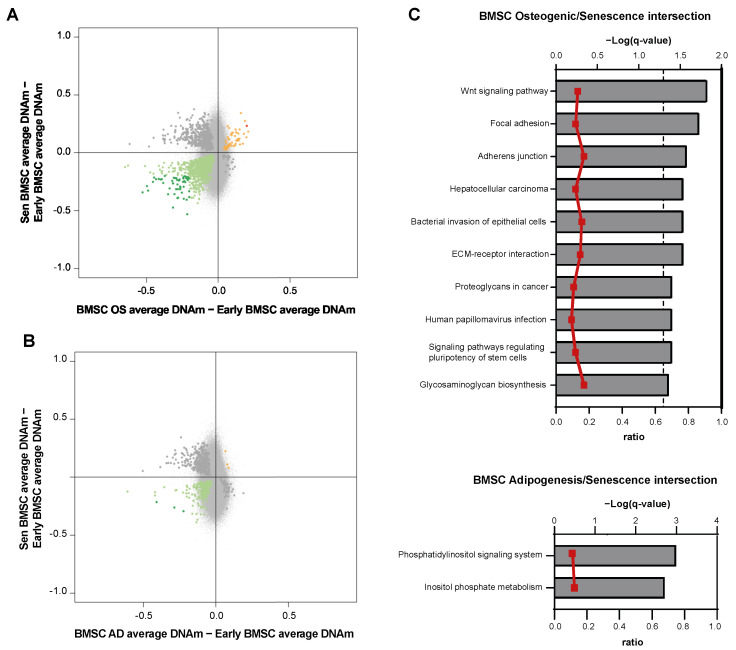
(**A**,**B**) Scatterplots comparing DNAm differences induced by (**A**) osteogenic (OS) and (**B**) adipogenic (AD) commitment vs. replicative senescence (Sen) of BMSCs. DMPs (FDR < 0.05) with hypo- and hyper-methylation in both the comparisons are indicated in green and red, respectively; DMPs with an absolute DNAm difference >0.20 are highlighted with darker green and red colors. (**C**) KEGG pathways significantly enriched in genes containing DMPs that are common between BMSC osteogenic (upper panel), adipogenic (lower panel) commitment, and replicative senescence. Pathways are ranked according to the significance of enrichment (grey bars, upper *y*-axis). Ratios referring to the proportion of targeted genes related to the total number of genes in each pathway are displayed (red line graph, bottom *y*-axis).

We finally investigated the relationship between DNAm changes occurring in BMSC differentiated into osteoblasts or adipocytes and in senescent BMSCs. For both differentiated cell types, DMPs showed a significant overlap with those associated to senescence (Fisher’s exact test <0.05): 1544 probes were differentially methylated in both osteogenic differentiation and senescence, while 540 in adipocytes (Supplementary Files S4 and S5). However, when we considered the direction of DNAm changes, we noted that DMPs associated to osteogenic differentiation tended to be more concordant with senescence-associated DMPs with respect to those identified in adipocytes: in the intersection between osteogenic-associated DMPs and senescence-associated DMPs, 58% of DMPs showed the same direction of change (mainly hypomethylation), while in the case of adipogenic-associated DMPs, only 32% of DMPs were concordant with those shared with senescence (Figure 5A,B). Pathway analysis performed on the DMPs at the intersections between MSC senescence and differentiation revealed a significant enrichment of 10 pathways for the senescence-osteogenesis intersection and 2 pathways for the senescence-adipogenesis intersection (Figure 5C).

## 4. Discussion

Here, we performed a genome wide DNA methylation analysis of proliferating and senescent human BMSCs and of BMSCs induced to differentiate into adipocytes and osteoblasts. We also compared the senescence-associated DNAm changes of BMSCs with endothelial (HUVECs), to identify a putative common methylation signature to be identified as a component of the replicative senescence program. Moreover, for the first time, we compared the methylation of senescent BMSCs and differentiated cells, in order to explore possible contact points between adipogenesis, osteogenesis, and replicative senescence.

As expected, we found that BMSCs and HUVECs are overall endowed with distinct CpG methylation patterns, with BMSCs showing a global hypomethylation compared to HUVECs. Despite the differences due to cell origin, a substantial overlap of DMPs was found in HUVEC and BMSC senescent cells, with a high concordance in terms of direction of the change. Indeed, both BMSC and HUVEC replicative senescence was accompanied by a genome wide hypomethylation. This observation is in agreement with the well described global hypomethylation associated with aging [24] and cell senescence [13,14,17], which tends to involve mainly CpG sites in interspersed and repetitive sequences highly methylated in cell at early passages. In this regard, it has been proposed that senescence-associated DNAm changes occur in a highly reproducible fashion in primary cell types, including BMSCs and HUVECs, thus providing a potential biomarker reflecting the state of cellular aging [25]. It is still debated whether this DNAm changes are associated with the acquisition of functional changes in senescent cells. Notably, we observed that albeit the number of DMPs (about 250,000) in senescent BMSCs and HUVECs was comparable, the latter exhibited more pronounced DNAm changes greater variability in terms of DNAm changes. DNAm changes have been historically associated with the establishment of a metabolic memory in endothelial cells exposed to hyperglycemic conditions [26]. Our data support the hypothesis that HUVECs—more than BMSCs—are capable of undergoing significant epigenetic changes associated with the acquisition of the replicative senescence phenotype, which can be fostered by metabolic derangements such as those observed in age-related diseases.

We performed a KEGG pathway enrichment analysis on genes carrying DMPs with the same direction of methylation change in HUVECs and BMSCs and retrieved 16 common pathways, including Insulin resistance, Molecule adhesion, and Wnt/β-catenin signaling. Both cell lineages are invariably involved in insulin signaling. Furthermore, aging mediates a series of DNAm changes in BMSC-derived adipocytes, which result in disrupted adipokine synthesis and secretion, predisposing to the progression of insulin resistance. Moreover, adipocytes developed from senescent progenitors exhibit reduced adipogenic capacity and impaired glucose tolerance which is further affected by the burden of proinflammatory cytokines associated with the SASP [27]. In the context of endothelial cell senescence, DNAm has been regarded as a principal mediator of the metabolic memory linked to long lasting insulin resistance and responsible for the development of atherosclerotic vascular disease in type 2 diabetes [28].

It is widely accepted that adhesion molecules, including vascular cell adhesion molecule-1 (VCAM-1), intercellular adhesion molecule-1 (ICAM-1), and E-selectin, are up regulated in senescent vascular endothelium and play a critical role in inflammatory leukocyte recruitment and migration in the subendothelial region [29,30]. Intimal leukocyte accumulation represents a fundamental step in atherosclerosis pathogenesis. When exposed to proinflammatory stimuli, also BMSCs are able to upregulate the expression of adhesion molecules—ICAM1 and VCAM1 among others [31]—although their importance has not been fully evaluated. Similarly to leukocytes, BMSCs exploit adhesion molecules to reach damaged tissues, the so called “homing” of mesenchymal stem cells which is gaining particularly attention in the field of regenerative medicine [32,33].

The Wnt/β-catenin signaling pathway is widely investigated in MSC biology as the major driver of osteogenic differentiation. Interestingly, a growing body of evidence supported its involvement also in MSC senescence, through the activation of DNA damage response and reactive oxygen species production [34,35]. Notably, Wnt/β-catenin is the most enriched pathway when considering DMPs shared by osteogenic differentiation and BMSC senescence, confirming also at the DNAm level the dualistic role of this signaling which is far from being elucidated and warrants future investigation [36,37,38]. In endothelial cells Wnt/β-catenin signaling has been associated with physiological and pathological angiogenesis [39], and with the acquisition of a dysfunctional phenotype mediated by the activation of the stress adaptor p66(Sch) [39].

A previous meta-analysis on a collection of Infinium 450 K methylation arrays performed on human subjects revealed that aging associated DNAm changes were significantly enriched in pathways involved in muscle biogenesis and neuronal signaling, which show a high degree of interindividual variation with increasing age [40]. In line with these findings, here we showed that most of the enriched pathways of DMPs showing a concordant differential DNAm in senescent HUVECs and BMSCs are related to neurotransmission and, to a lesser extent, to muscle function.

As regards differentiation, in both adipogenesis and osteogenesis we observed a general demethylation of CpG sites compared with undifferentiated BMSCs with a higher number of DMPs in osteogenesis. The more pronounced DNAm changes in osteogenesis resulted in the identification of 30 enriched pathways when compared to undifferentiated BMSCs, while only 2 pathways were identified in adipocytes when compared to undifferentiated ones. Interestingly, all the shared DMPs in adipogenesis and osteogenesis displayed the same direction of DNAm with respect to undifferentiated BMSCs and KEGG pathway enrichment analysis on genes carrying concordant DMPs in AD and OS identified Inositol phosphate metabolism and Phosphatidylinositol signaling system. Inositols and inositol phosphates (IPs) are well-known to be important for cell signaling regulating energy homeostasis and anti-oxidant and anti-inflammatory activities. IPs are well studied in adipocytes where these molecules are involved in energy metabolism, in insulin signaling and in browning [41]. Inositol phosphates were less investigated in osteogenesis. A recent study showed that the lipid kinase phosphatidylinositol-4-phosphate 5-kinase type 1 gamma (Pip5k1c) is essential in MSCs for osteoblast development [42]. The specific retrieval of inositol phosphate pathways when considering the intersection of adipogenesis- and osteogenesis-related DNAm changes supports the importance of this intracellular signaling pathway in the differentiation of BMSCs.

Finally, when comparing differentiated BMSCs with senescent ones, we found out that the 58% of DMPs were in common and shared the same direction of DNAm change (mainly hypomethylation) between osteogenesis and senescence, while in the case of adipogenesis-associated DMPs, only 32% of DMPs were concordant with BMSC senescence. Beyond Wnt signaling, the intersection of OS and Senescence revealed 10 common pathways, including signaling pathways regulating pluripotency of stem cells, and only two pathways, i.e., Inositol phosphate metabolism and Phosphatidylinositol signaling system, that were shared between DMPs of AD and senescent cells and, as abovementioned, characterize the adipogenesis DNAm pattern. The wider concordance of methylation between senescent mesenchymal cells and BMSC-derived osteoblasts could be counterintuitive, since adipose tissue tends to accumulate in aged bone marrow. However, the relative contribution of epigenetic modifications to the differentiative potential and functional phenotype of BMSCs still needs extensive investigation. Remarkably, a differential methylation of genes involved in bone formation and remodeling has been reported by genome-wide association studies performed on subjects with reduced bone mineral density, prompting the hypothesis that assessing the DNAm status of selected genes could provide useful biomarkers to identify individuals at high risk of developing osteoporosis [43]. Interestingly, altered methylation patterns have been highlighted also in BMSCs from patients with hematological malignancies, including acute myeloid leukemia [44], multiple myeloma [45], and myelodysplastic syndromes [46], and the therapeutic response to demethylating agents such as azacytidine has been associated with the restoration of BMSC physiological phenotype [46]. Altogether, this evidence reinforces the role of epigenetic modifications as critical mediators of the crosstalk between BMSCs and the other compartments of the BM microenvironment in multiple disease states.

The present study has limitations that need to be addressed. The study design does not allow us to draw any mechanistical conclusion on the role of methylation on our cellular conditions. Moreover, to date we have not performed RNA sequencing analysis on BMSCs and HUVECs which could allow us to associate methylation with gene expression data. Finally, due to the great heterogeneity of BMSCs we cannot generalize our data and further studies are needed on BMSCs derived from older donors. However, our preliminary study approached for the first time to DNAm as a tool to describe, and possibly predict, the fate of BMSCs by comparing the most relevant differentiation pathways, i.e., adipogenesis and osteogenesis, with replicative senescence.

Overall, we can conclude that BMSC senescence and differentiation are accompanied by a genome-wide DNA hypomethylation which, however, involves different sets of CpG probes, thus allowing a clear separation of the paths that these cells could undertake. DNA methylation analysis could help the comprehension of how the differentiation potential of mesenchymal stem cells could be influenced by the senescence status. Future mechanistic studies are needed to translate these findings into the pathobiology of age-related bone disease.

## Data Availability

The data presented in this study are available on request from the corresponding author.

## Supplementary Materials

The following supporting information can be downloaded at: https://www.mdpi.com/article/10.3390/cells12020285/s1, Figure S1: Boxplots of DNA methylation values of InfiniumEPIC probes in early and senescent BMSC and HUVEC and in BMSC induced to differentiate into adipocytes and osteoblasts. Figure S2: The scatterplots compare mean DNAm levels in either BMSCs and HUVECs at early and late passages. Figure S3: Enrichment of senescence-associated hypo- and hypermethylated CpGs across genomic features. Figure S4: Scatterplots comparing DNAm values of early BMSCs vs. osteogenic- or adipogenic-committed BMSCs. Figure S5. Enrichment of commitment-associated hypo- and hypermethylated CpGs across genomic features. Supplementary File S1: Differentially methylated probes (DMPs) between late- and early-passage BMSCs. Supplementary File S2: Differentially methylated probes (DMPs) between late- and early-passage HUVECs. Supplementary File S3: KEGG-enriched pathways based on DMPs in senescent HUVECs, BMSCs, BMSC-derived osteoblasts and adipocytes. Supplementary File S4: Differentially methylated probes (DMPs) between BMSC-derived osteoblasts and undifferentiated BMSCs. Supplementary File S5: Differentially methylated probes (DMPs) between BMSC-derived adipocytes and undifferentiated BMSCs. Supplementary File S6: Differentially methylated probes (DMPs) between BMSC-derived osteoblasts and adipocytes.
