# Supplementary material for: Genome-Wide Methylation Changes Associated with Replicative Senescence and Differentiation in Endothelial and Bone Marrow Mesenchymal Stromal Cells
Source: Cells. 2023 Jan 11;12(2):285. doi: 10.3390/cells12020285 (PMC9857206; doi:10.3390/cells12020285)
Supplement: Supplementary file 1 [file cells-12-00285-s001.zip › Supplementary Figures.pdf]

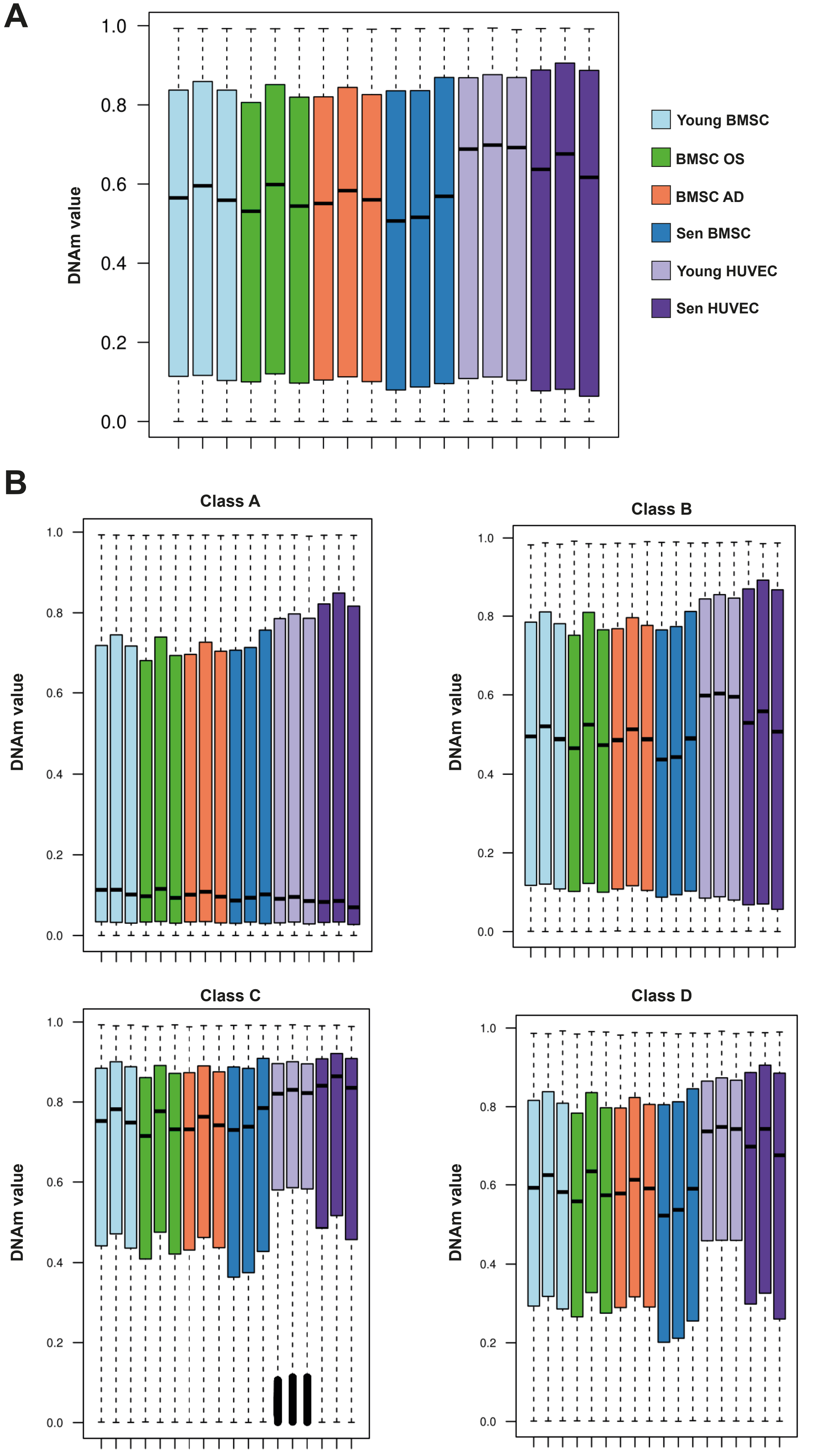

Figure S1. Boxplots of DNA methylation values of InfiniumEPIC probes in early and senescent BMSC and HUVEC and in BMSC induced to differentiate in Adipocytes (AD) and osteoblasts (OS). All the celllualr conditions are in triplicates. The probes were divided in four groups according to their genomic location, i.e. probes in CpG islands/shores/shelves in a gene (Class A), in CpG islands/shore/shelves not in a gene (Class B), not in CpG islands/shores/shelves in a gene (Class C), and in open sea (Class D).

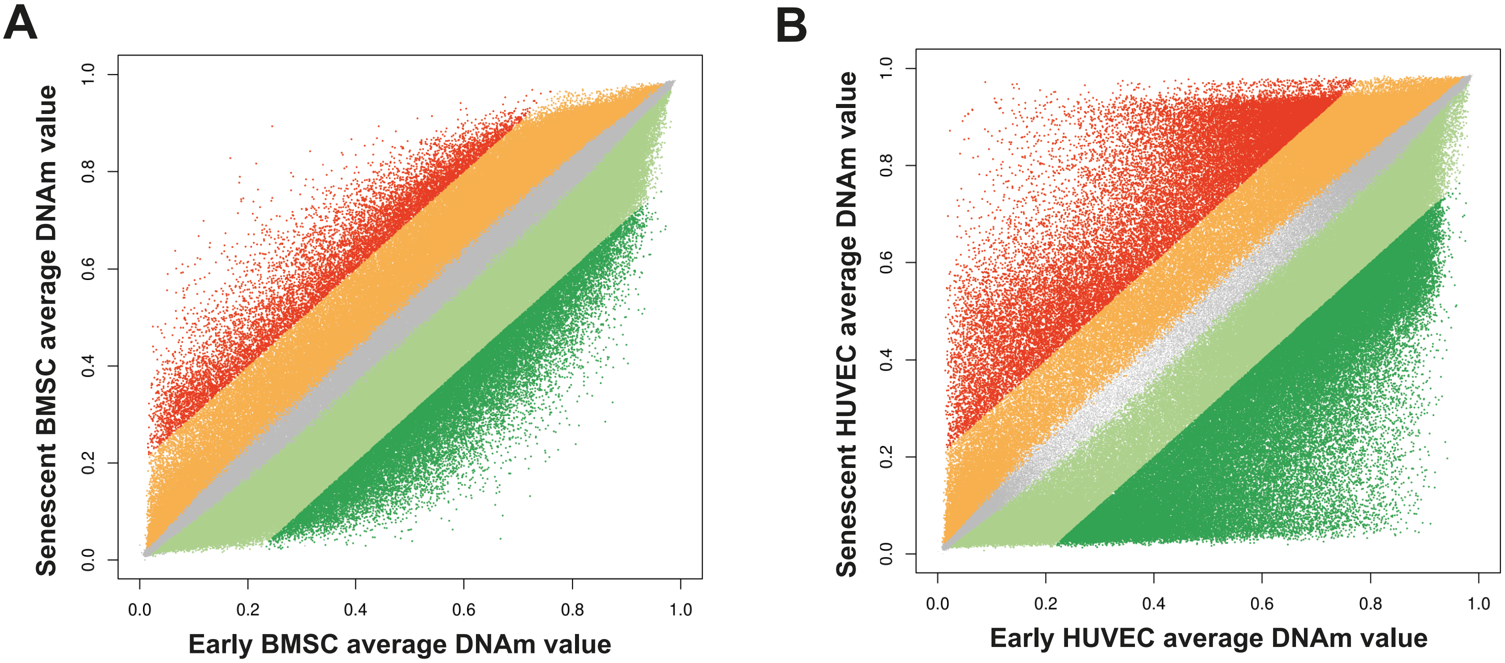

Figure S2. The scatterplots compare mean DNAm levels in either BMSCs and HUVECs at early and late passages. CpGs with significant senescence-associated (20% change in DNAm level and adjusted P-value < 0.05). hypo (dark green)- and hypermethylation (red) are indicated.

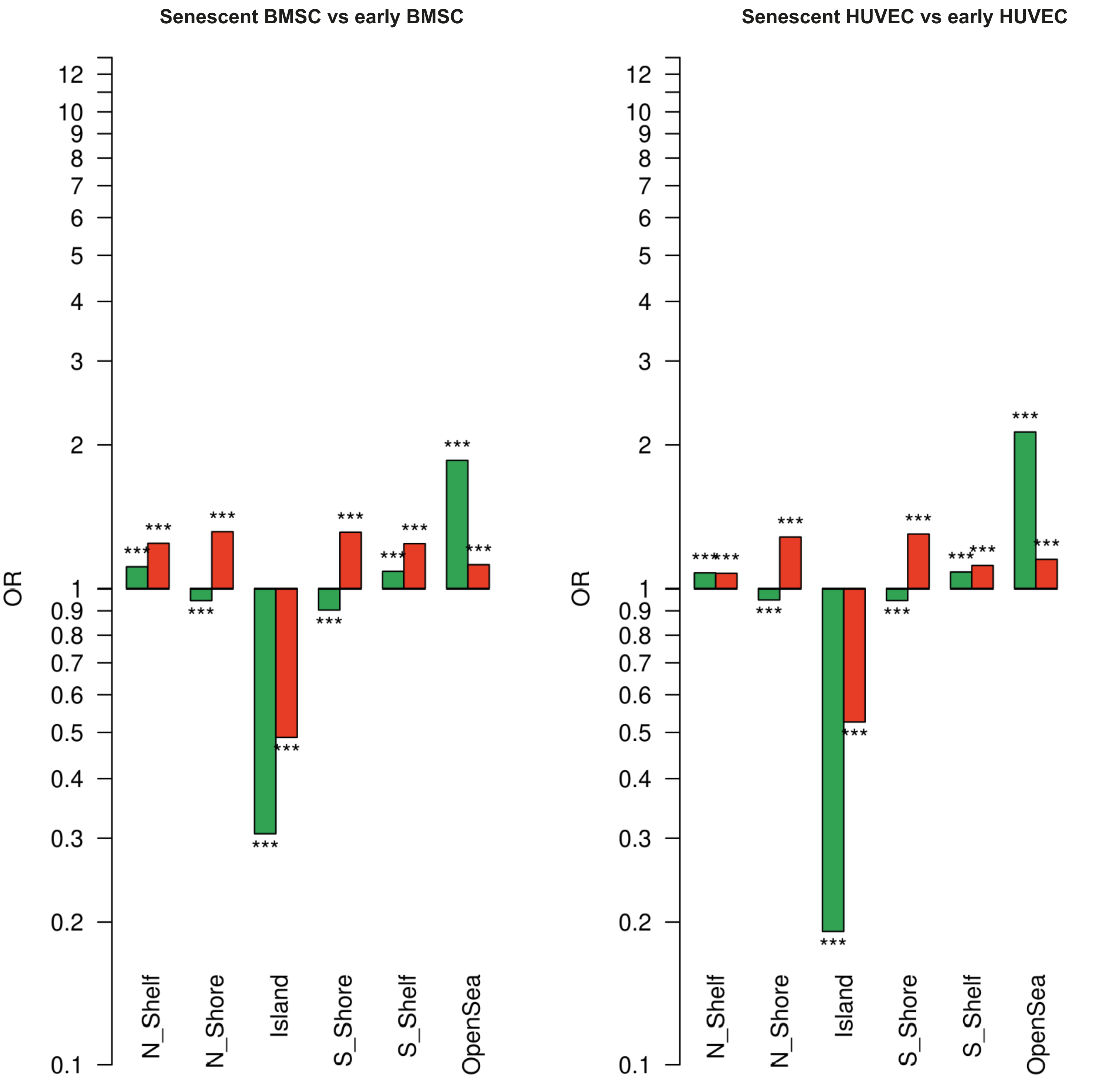

Figure S3. Enrichment of senescence-associated hypo (green)- and hypermethylated (red) CpGs across genomic features. P-values: *** *p* < 0.001 vs. expected.

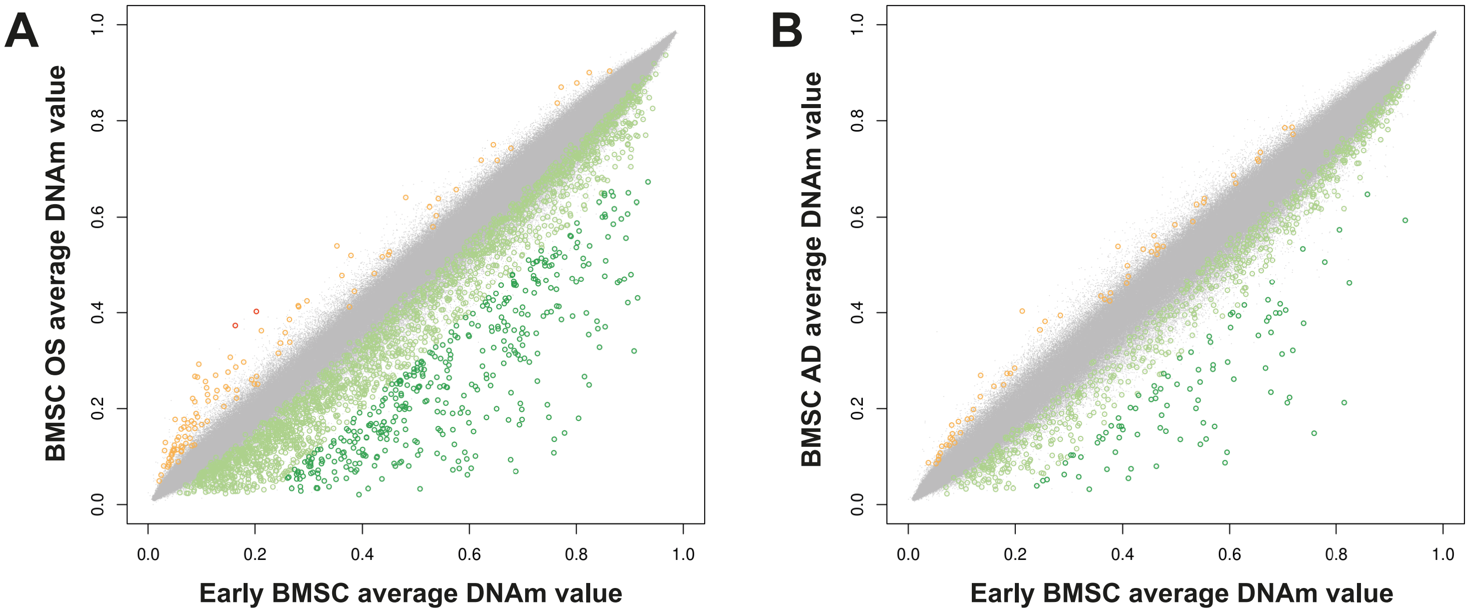

Figure S4. Scatterplots comparing DNAm values of early BMSCs vs osteogenic- (OS, A) or adipogenic (AD, B) committed BMSCs. CpGs (DNAm difference >0.20 and FDR < 0.05) with concordant hypo (green)- and hypermethylation (red) are indicated.

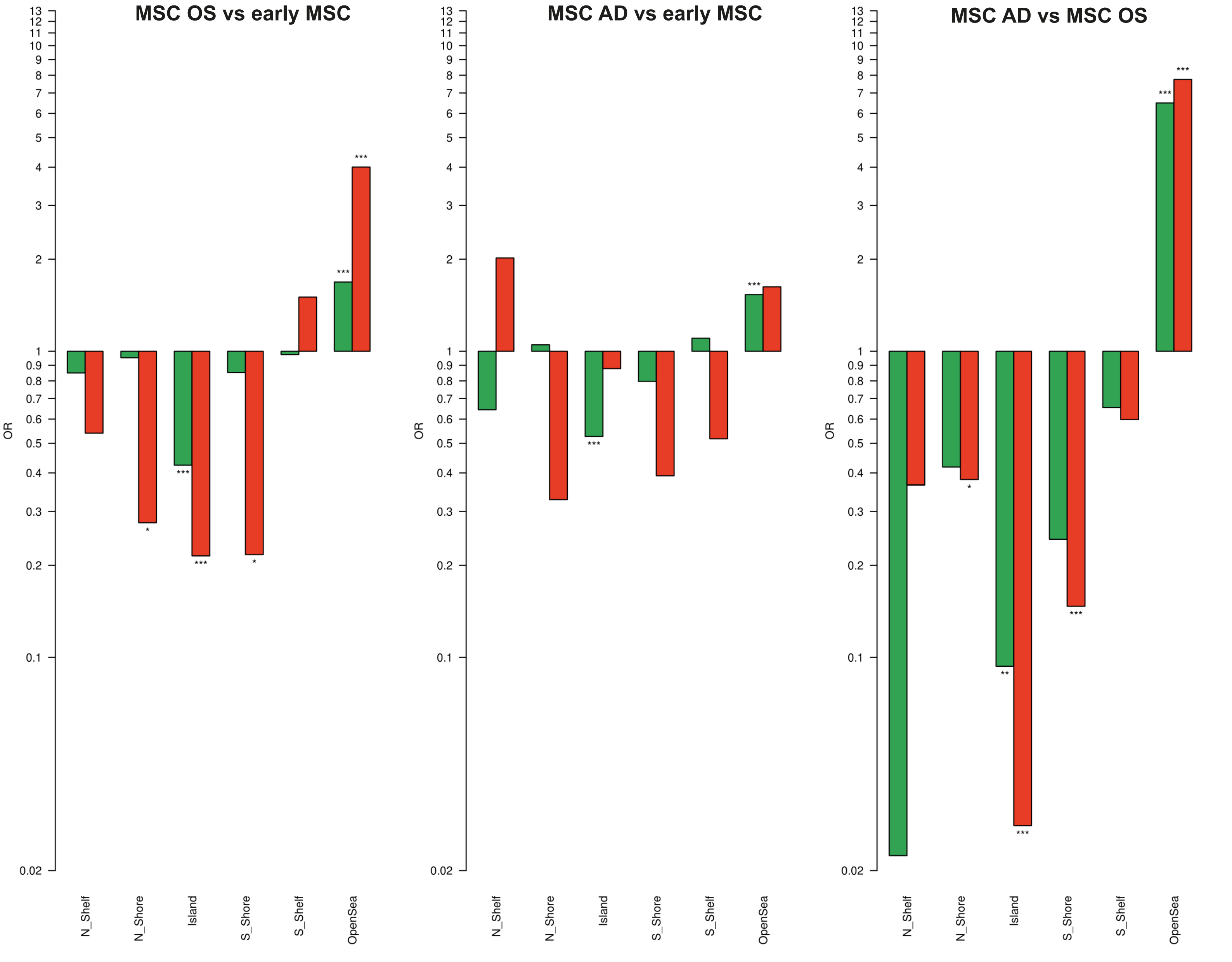

Figure S5. Enrichment of commitment-associated hypo (green)- and hypermethylated (red) CpGs across genomic features.
*, $p < 0.05$ *** $p < 0.001$ vs. expected.
